# Paroxysmal Atrial Fibrillation (PFA) Detection With Cardiac Monitoring Devices Implanted by Neurologists in Patients With Embolic Strokes of Undetermined Source (ESUS)

**DOI:** 10.7759/cureus.51168

**Published:** 2023-12-27

**Authors:** Patricio S Espinosa, Patricio H Espinosa del Pozo, Nicolas F Andrade, Kettia Alusma-Hibbert

**Affiliations:** 1 Neurology Department Stroke Division, The Espinosa Neuroscience Institute, Boca Raton, USA; 2 Neuroscience, Universidad Central Del Ecuador, Quito, ECU; 3 Biological Sciences Department, Webster University, St. Louis, USA

**Keywords:** neurologist, paroxysmal afib, loop recording device, esus, stroke

## Abstract

Introduction

Ischemic stroke is among the leading causes of death and disability. Approximately 50% of cryptogenic strokes are embolic strokes of undetermined source (ESUS). The most common cause of ESUS is atrial fibrillation. Therefore, the detection of atrial fibrillation with long-term implantable devices is needed. Neurologists are directly involved with acute and post-acute stroke care and have direct access to the management of stroke patients. Therefore, there is a need for neurologists to recommend, implant, and monitor cardiac implantable devices in patients with ESUS.

Methods

From November 2022 to October 2023, our group implanted 32 ESUS patients with Confirm Rx™ insertable cardiac monitors (Abbott, USA). Atrial fibrillation detection was supervised and monitored daily.

Results

In 24 months, atrial fibrillation was detected in 12.5% of patients (four patients), sinus bradycardia in 6.25% of patients (two patients), paroxysmal supraventricular tachycardia in 9.4% of patients (three patients), and asystole in one patient.

Conclusion

Our study shows that neurologists involved in the treatment of stroke care can safely implant, monitor, and detect atrial fibrillation accurately. Our rate of detection of atrial fibrillation in patients with ESUS was 12.8%, which is consistent with prior studies.

## Introduction

Ischemic stroke is among the leading causes of death and disability globally [1,2]. The etiology of stroke can not be determined after routine acute stroke evaluation in about 30% of cases [2,3]. This subgroup of ischemic stroke patients where the cause is not found is called cryptogenic stroke. About 50% of cryptogenic strokes are embolic strokes of undetermined source (ESUS). The most common cause of ESUS is atrial fibrillation (AF). Atrial fibrillation can lead to the formation of emboli that can result in large vessel occlusion, causing significant disability to a patient [3,4]. A type of AF is paroxysmal atrial fibrillation (PAF), which is intermittent AF and resolves on its own. Although PAF is intermittent, the risk of emboli formation still can occur [4,5]. Prevention of emboli formation is typically treated with oral anticoagulant therapy. Evidence has shown that treating ESUS with anticoagulation without cardioembolic origin was not superior to aspirin [5]. Moreover, patients on anticoagulation medicines have a higher risk of bleeding, therefore, the need for cardiac monitoring [5,6]. The detection of PAF with long-term implantable cardiac monitoring devices is needed to determine if anticoagulation is needed. Implantable loop recorders (ILRs) are implantable cardiac monitors that can be monitored for up to three years and are considered safe and sensitive in the detection of PAF. Studies have shown that ILRs can detect PAF in up to 30% of patients with ESUS [5-7].

Traditionally, the implantation of ILRs has been led by cardiologists and electrophysiologists, which can result in delay of implantation, detection, and communication with stroke neurologists and patients. On the other hand, neurologists are the primary care providers involved with stroke care and have continuous access to the management of stroke patients. For this reason we believe the need for neurologists to recommend, implant, and monitor patients with ESUS [7]. We report the data of our patients with ESUS where PAF was detected using neurologist-led ILRs.

## Materials and methods

Demographics

The study comprised a total of 32 patients, including 23 females and nine males. The median age was 73 years, with a standard deviation of 14 years. The age range spanned from 36 to 90 years (Table 1).

**Table 1:** The key demographic information from the study

Total Patients	Females	Males	Median Age (Y)	Standard Deviation (Y)	Age Range (Y)
32	23	9	73	14	36 - 90

Criteria used for IRL implantation

Patients with acute neurological deficits were diagnosed with embolic strokes by magnetic resonance imaging (MRI) of the brain and the etiology of the stroke was not found after appropriate cardioembolism workup. The diagnostic studies used in the stroke workup included: computed tomography (CT) of the brain, CT and/or MRI angiography of the head and neck, carotid duplex ultrasound scans of the carotid arteries, transthoracic and/or transesophageal echocardiography with bubble study to rule out Patent Foramen Ovale (PFO), routine 12 lead electrocardiogram (EKG), and external cardiac telemetry monitoring for at least 72 hours. Once the workup was completed and the etiology of the embolic stroke was not found, the diagnosis of ESUS was made. Other diagnoses were not eligible for ILRs, which were the only specific exclusion criteria.

Once the indication for ILRs was made, eligible patients with ESUS were informed about the benefits, risks, and goals of the treatment plan associated with the in-office procedure. Informed consent for the procedure was obtained. The ESUS patients had an ILR implanted by our stroke neurologist in the outpatient neurology office setting. Implantation was performed with an aseptic technique and topical anesthesia with a small skin incision in the left parasternal third to fourth intercostal space. The device implanted in all of our patients was the Abbott Confirm Rx™ insertable cardiac monitor (Abbott, USA). This device has automatic data transmission through Bluetooth and the Merlin Abbott phone application. The patients were instructed and trained in the use of the phone application, connectivity, and daily transmissions. The patients also had accessibility to office staff to help triage concerns following the procedure. 

The Abbott Confirm Rx ™ insertable cardiac monitor uses the AF algorithm: a 4-step “R R” variability (Markov chain variance, 64 beat window), onset fast/slow, “P” wave detection, secondary “P” wave discriminator (Dynamic thresholds, review of prior 30 seconds). An automatic algorithm detection would notify each AF episode, regardless of duration, for manual analysis. AF episodes were verified one by one manually by our experienced neurologist. AF was diagnosed with a duration of at least 30 seconds. We used a board-certified electrophysiologist to confirm all AF cases. Once the diagnosis of AF was made, patients were notified and had immediate follow-up to start on anticoagulation therapy and referred to electrophysiology for further recommendations. IRLs were still continued to be monitored by a stroke neurologist. IRB approval was waived due to the retrospective nature of the study, involving a deidentified chart review of records.

## Results

From November 2021 to October 2023, our group implanted 32 patients with ESUS with Abbott Confirm Rx™ insertable cardiac monitors. Atrial fibrillation (AF) detection was supervised and monitored daily. In 24 months, PAF was detected in 12.5% of patients (four patients), Sinus bradycardia in 6.25% of patients (two patients), paroxysmal supraventricular tachycardia in 9.4% of patients (three patients), and pause of 7 seconds in one patient. 

**Figure 1:**
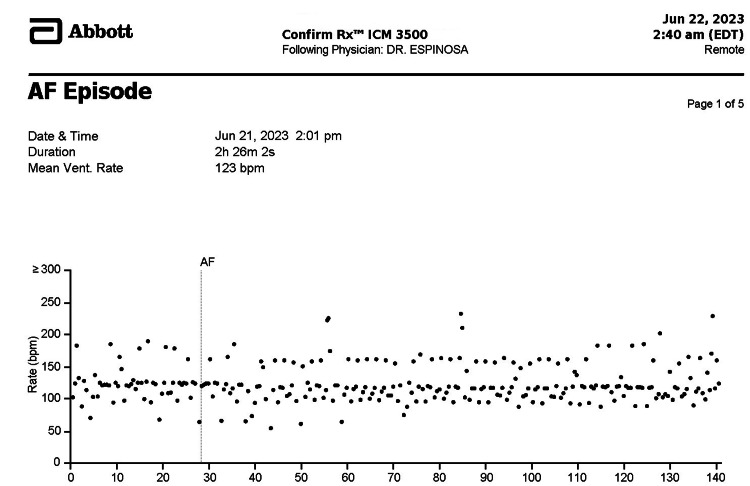
The report of a patient with ESUS diagnosed with AF Note the trigger after 30 seconds and the sustained AF for over 140 seconds. HR ranges from 50 - 200 irregular beats per minute. ESUS: Embolic strokes of undetermined source; AF: Atrial fibrillation; HR: Heart rate. Confirm Rx^TM ^ICM 3500 is the Insertable Cardiac Monitor (Abbott, USA).

Figure 1 demonstrates a patient with ESUS who was diagnosed with AF following implantation of ILR. The figure shows in the Y-axis the heart rate in beats per minute and in the X-axis times in seconds. A vertical line label (AF) is the trigger of detection of AF at ~30 seconds and sustained AF for over 140 seconds. The heart rate ranged from 50 - 200 irregular beats per minute.

**Figure 2:**
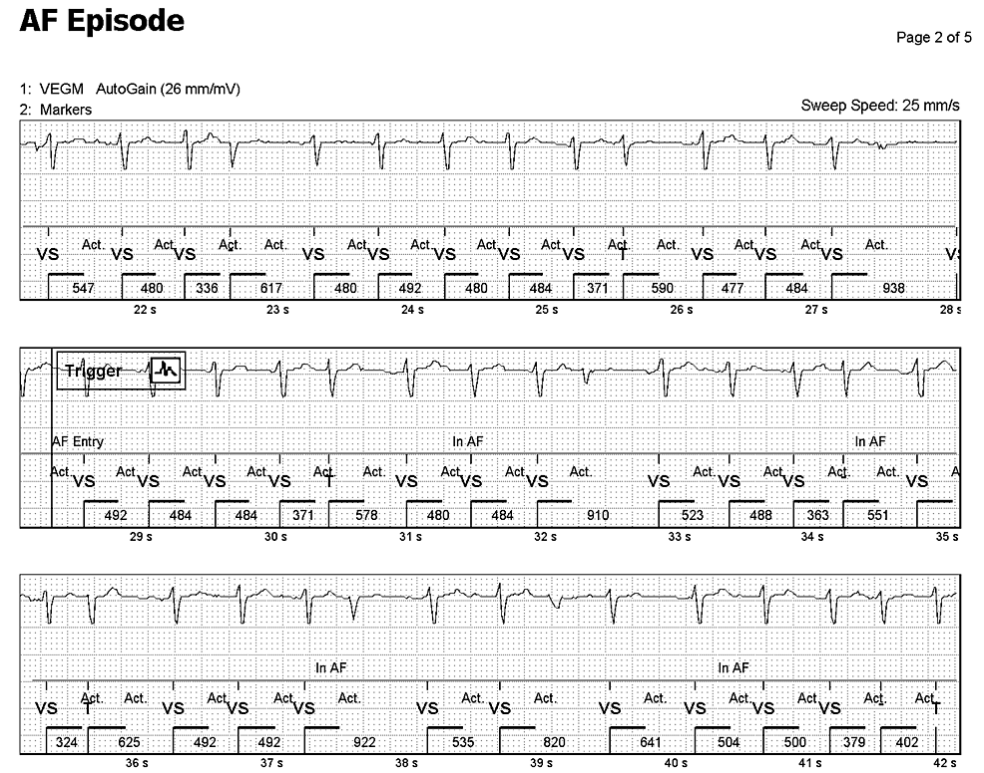
Single lead EKG tracing generated by the ILR algorithm showing sustained AF for over 30 seconds ILR: Implantable loop recorder

Figure 2 shows a single lead EKG tracing on the same patient, showing the trigger of the algorithm when the patient had sustained AF for over 29 seconds. Note the classic features of the AF that can be easily identified: irregularly irregular rhythm, variable ventricular rate, and the absence of P waves.

**Figure 3:**
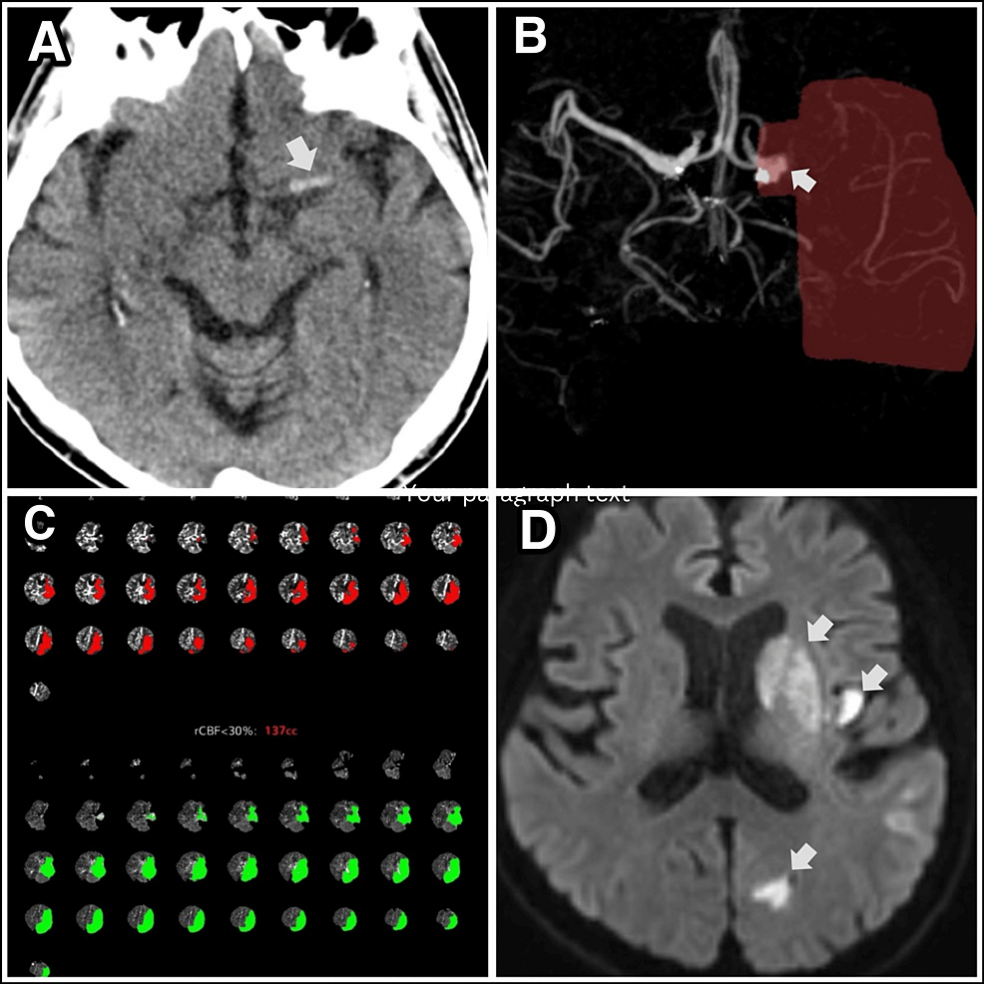
Imaging of a patient with an acute ischemic embolic stroke A) CT scan of the head shows a left hyperdense left middle cerebral artery (MCA) sign (arrow), B) Rapid ® artificial intelligence software showing the left MCA M1 clot (arrow), C) VizAi ® showing  Mismatch ratio 1.8 ( Tmax > 6sec- 251 mL, CBF <30%-137 mL). D) MRI of the brain DWI sequence showing restricted diffusion involving the left basal ganglia (top arrow), portions of the insular cortex on the left (middle arrow), and ischemia in the region of the left occipital lobe (bottom arrow). The patient had a fetal origin of the posterior cerebral artery( PCA); therefore, an infarction in the occipital lobe.

Figure 3 demonstrates acute stroke imaging of a patient with ESUS diagnosed with PAF one month after the ILR was implanted. Panel A) CT scan of the head, which shows a left hyperdense left middle cerebral artery (MCA) sign (arrow); panel B) shows the output of Rapid ® artificial intelligence (AI) software (RapidAI, San Mateo, CA) demonstrating an image of the left MCA M1 clot, with lack of blood flow with an estimation of ischemia area at risk due to lack of blood flow; panel C) shows the output VizAi ® AI (Viz.ai, San Francisco) showing ischemic core (red) versus penumbra (green) with a mismatch ratio 1.8 ( Tmax > 6sec- 251 mL, CBF <30%-137 mL) which is another illustration of area for risk of further ischemia to brain; panel D) MRI of the brain with diffusion-weighted imaging (DWI) sequence showing restricted diffusion involving the left basal ganglia, portions of the insular cortex, and the occipital lobe. The patient had a fetal origin of the posterior cerebral artery (PCA); therefore, area of ischemia in the PCA region.

## Discussion

Our data shows that neurologists can lead the evaluation, implantation, and monitoring of ILRs in patients with cryptogenic stroke. Our detection rate of AF was 12.8% in patients with ESUS, which was similar to previous studies [4]. Our data adds to the body of evidence that stroke neurologists can safely, appropriately, and effectively monitor and treat ESUS patients with AF [3,4,7].

Immediate detection of PAF is crucial in the treatment of embolic acute ischemic strokes [6]. The ability of stroke neurologists involved in acute stroke management to implant and monitor ILRs improves stroke patient outcomes. All patients with transient ischemic attacks (TIA) and ischemic strokes are typically monitored in telemetry for at least 72 hours at minimum in the acute stroke setting. After this period, patients diagnosed with ESUS should be implanted with ILRs if appropriate. Studies have shown that the AF in this group of patients typically occurs within 30 to 60 days from stroke onset. Therefore, non-implantable short-term cardiac monitors may miss episodes of AF, resulting in preventable strokes [4-8].

Currently, the standard practice is to consult cardiology to implant ILRs once ESUS is confirmed. In the inpatient setting, neurologists consult cardiology or electrophysiologist (EPS) services for ILRs, however, this creates varying logistical challenges that negatively affect the patient care. One of the challenges that we have identified is the delay in care. Many times during weekends and holidays, patients with ESUS have to have prolonged hospital stays waiting for implantation or be discharged without implantation of ILRs. This causes a break in effectively delivering comprehensive acute stroke care. Neurologists, on the other hand, have direct contact with patients with stroke during the initial evaluation, acute treatment, and management throughout the inpatient stay and during outpatient follow-up. This flow ensures consistency in the delivery of care, which impacts positively by reducing costs and improving stroke care. Therefore, neurologists-led implantation of ILRs is cost-effective, efficient, and safe for patients.

In the outpatient setting, if patients with ESUS need an ILR, the current practice in the majority of neurology practices in the United States of America (USA) is to send a referral to cardiology for a consult for loop recorder implantation. This creates another logistical challenge, as the patients have to find cardiologists or EPS who accept their healthcare plan and can evaluate and implant loop recorders in a timely fashion. Typically, the patient will need a new patient consultation. This may not be for several months, and after the consultation, the cardiologist or EPS has to schedule the patient for another visit to implant. This already delays the patient and potentially puts them at risk for further strokes that could be preventable if delay of care did not occur. On the other side, neurologists who are taking care of stroke patients have already established relationships and can implant loop recorders efficiently.

For the past 10 years, cardiologists and EPS have led the implantation of ILRs in the USA. Our neurology practice continues to provide stroke management care for these patients who have had ILRs implanted by cardiologists or EPS. To date, this has not been a reliable way of getting consistent reports of the cardiac monitoring of patients implanted outside of our practice. Most of the time, patients have a hard time following up, contacting cardiology, and obtaining their cardiac monitoring reports. Moreover, these patients have a difficult time understanding how the device works and how the data is transmitted. This creates uncertainty about whether the ILRs are being monitored accurately. Therefore, the need for a change in the practice is to have stroke neurologists lead the implantation of ILRs in acute stroke management. 

We also encounter several times that due to the lack of training in stroke, there is overuse and over implantation of ILRs. Training in neuroimaging and stroke is crucial and only neurologists with stroke training can differentiate an embolic stroke from a thrombotic stroke, or a stroke that is embolic in origin due to watershed, or an embolic artery-to-artery stroke. Not all patients with an embolic stroke should be implanted with an ILR. Therefore, there is a need to change the practice for stroke neurologists to lead the implantation of ILRs in patients with ESUS [7-9].

While AF detection changes therapeutic decisions imminently, the causality of AF and ESUS remains controversial to some extent [10-12]. In our opinion, aggressive treatment of potential stroke risk factors can decrease stroke risk and disability. In addition, it is not clear what duration is needed for an AF episode to cause an embolic stroke. The Abbott Confirm Rx™ insertable cardiac monitor is programmed to detect AF after 30 seconds of AF, other devices are set up to detect after 120 seconds. To date, studies have been inconclusive in the timing needed for AF; however, it is our opinion that for patients with ESUS, one event of 30 seconds of PAF is enough to initiate anticoagulation with close follow-up and monitoring [12-15].

One of the weaknesses of our study is the relatively small sample size, and it was conducted at a single center. As a result, it is essential to approach our conclusions with a measure of caution and recognize the potential limitations imposed by the limited number of participants.

## Conclusions

Our study shows that neurologists can and should lead the implantation and monitoring of ILR in the setting of acute ischemic embolic stroke. Monitoring of ILRs by neurologists is cost-effective and beneficial for the treatment of patients with acute ischemic stroke. The detection rate of PAF in patients with ESUS in our study was 12.8%, which is consistent and similar to prior studies.
